# Development and Characterization of Monoclonal Antibodies for the Mycotoxin Citreoviridin

**DOI:** 10.3390/toxins11110630

**Published:** 2019-10-30

**Authors:** Chris M. Maragos, Yosuke Uchiyama, Naoki Kobayashi, Fumichika Kominato, Yoshiko Sugita-Konishi

**Affiliations:** 1 Mycotoxin Prevention and Applied Microbiology Research Unit, Agricultural Research Service, U.S. Department of Agriculture, Peoria, IL 61604, USA; 2 Department of Food and Life Science, Graduate School of Life and Environmental Sciences, Azabu University, Sagamihara, Kanagawa 252-5201, Japan; de1701@azabu-u.ac.jp (Y.U.); n-kobayashi@azabu-u.ac.jp (N.K.); y-konishi@azabu-u.ac.jp (Y.S.-K.); 3 Shinsei Chemical Company Ltd., Ibaraki, Osaka 567-0085, Japan; kominato@schem.jp

**Keywords:** citreoviridin, antibody, immunoassay, rice

## Abstract

Citreoviridin (CTV) in an inhibitor of mitochondrial ATPase that has been isolated from molded yellow rice and linked to the human disease Shoshin-kakke (acute cardiac beriberi). The disease results from a deficiency of thiamine, however, purified CTV can reproduce the symptoms in experimental animals. The link between CTV and Shoshin-kakke has been difficult to resolve, in part because cases of the disease are rare. In addition to rice, CTV has been found in maize, pecan nuts, and wheat products. A method to screen for CTV and its geometric isomer, iso-CTV, in commodities was developed, based upon the isolation of two novel monoclonal antibodies (mAb). In an antigen-immobilized competitive enzyme-linked immunosorbent assay format (CI-ELISA), the observed IC_50_s for CTV were 11 ng/mL and 18 ng/mL (mAbs 2-2 and 2-4, respectively). The assays were relatively tolerant to methanol and acetonitrile, which allowed their application to the detection of CTV in spiked polished white rice. For quantification, a standard mixture of CTV and iso-CTV was used, along with matrix matched calibration. The dynamic range of the ELISA using mAb 2-4 was equivalent to 0.23 to 2.22 mg/kg in rice. Recoveries over the range of 0.36 to 7.23 mg/kg averaged 97 ± 10%. The results suggest that the mAb 2-4-based immunoassay can be applied to the screening of white rice for CTV. Both mAbs were also observed to significantly enhance the fluorescence of the toxin.

## 1. Introduction

In Japan in the years before World War I and continuing through the 1920s, there were human illnesses associated with consumption of moldy, yellow, rice. The illness, classified at the time as Shoshin-kakke (acute cardiac beriberi) decreased in incidence significantly around 1910, a fact attributed to increased inspection of rice by Japanese authorities [1]. The disease was related to beriberi, now known to be caused by thiamine deficiency. By 1930 Shoshin-kakke had almost completely disappeared from Japan [1]. Subsequent investigations in Japan led to the differentiation of the broad category of yellow-colored rice into five groups, of which four are caused by Penicillium spoilage and one by *Eurotium amstelodami* [2]. The four caused by Penicillia are each associated with a different species of fungus and a different causative agent. The type of yellow rice known as Ou-hen-mai is infested with *Penicillium citreonigrum* and has been associated with Shoshin-kakke. In 1964 the structure of the mycotoxin believed to be the causative agent (citreoviridin, CTV) was reported [3]. Another type of yellow rice is Citrinum yellow rice (Citrinum ou-hen-mai) where the causative fungus is *P. citrinum* and the associated mycotoxin is citrinin. In 2006–2008 an outbreak of beriberi occurred in the Maranhão state of Brazil. Despite the presence of a few samples contaminated with CTV, the cases appear to have been predominantly a result of thiamine deficiency, as many were reversed following administration of thiamine [4,5,6]. An excellent summary of the history of yellow rice and the classification of rice infested with fungi was provided by Kushiro [2].

The connection of Shoshin-kakke to moldy rice has been confounded by the multiple types of “yellow rice”, the low incidence of the disease in modern times, and the extent to which thiamine deficiency is required to produce symptoms. In research conducted in the 1960s and early 1970s, crude alcohol extracts of moldy rice were tested in 14 vertebrate species [1,7]. Symptoms included paralysis of the legs, vomiting, convulsions, and respiratory arrest [7]. Purified CTV given to mice, cats, and dogs reproduced these symptoms, with an LD_50_ of 20 mg/kg in mice [1]. Purified CTV given to mice and rats was lethal, with LD_50_s ranging from 3.6 to 11 mg/kg [7]. Reproducing symptoms of Shoshin-kakke with purified CTV was important for distinguishing intoxication due to consumption of yellow rice, from disease caused directly by thiamine deficiency. As such, Shoshin-kakke is considered to be a mycotoxicosis [2].

On the molecular level, CTV inhibits the mitochondrial adenosine triphosphatase (ATPase) [8,9]. When given to rats CTV altered the pattern of transketolase (EC 2.2.1.1) in liver, and in vitro experiments suggested an anti-thiamine effect of the toxin [10]. A mechanism that involves the exacerbation or causation of thiamine deficiency would be consistent with the involvement of CTV in Shoshin-kakke. Recently, the toxicokinetics of CTV was determined in swine [11]. Results suggested that following oral exposure, CTV was readily absorbed by swine, and slowly metabolized, with a half-life of 21 h [11].

**Figure 1:**
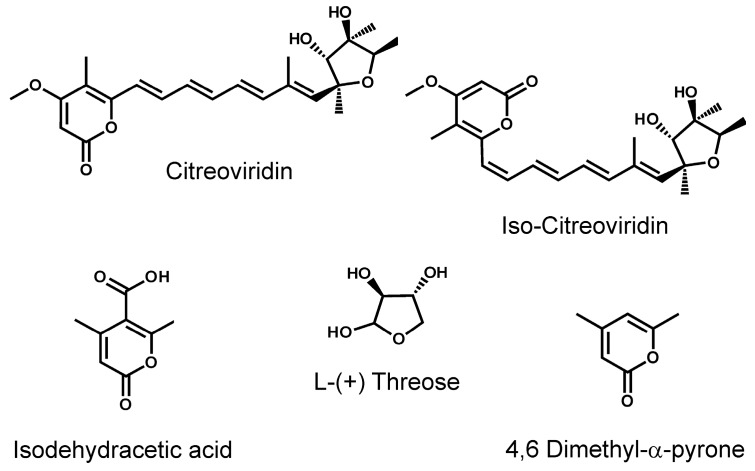
Structure of citreoviridin (CTV) and related compounds.

CTV (Figure 1) is produced by a variety of fungi including *P. citreonigrum*, *Aspergillus terreus*, and *Eupenicillium ochrosalmoneum* (actual identity *P. ochrosalmoneum*) [12,13,14]. The fungi that produce CTV were summarized recently by Peterson et al. [15]. While it is known primarily for its association with rice in Asia, CTV has been found in maize and pecan nuts in the United States [16,17]. It has also been found in rice and wheat-based products in Brazil [18] and in grain dust in Belgium [19].

The structure of CTV was first determined in 1964 [3]. Many of the physical properties of CTV were reported by Ueno and Ueno [7] and were summarized by Cole and Cox [20]. Purified CTV is bright yellow and, in methanol, has absorption maxima in the ultraviolet at 383 nm (ε 44,925), 294 nm (ε 24,725), 285 nm (ε 23,343), 238 nm (ε 10,383), and 203 nm (ε 15,388) [21]. Similar absorption maxima, but lower molar absorptivity coefficients were reported more recently, also in methanol: 387 nm (ε 31,590), 294 nm (ε 21,960), and 285 nm (ε 20,060) [13]. The latter also reported Fourier transform infrared (FTIR), ^1^H-, and ^13^C-nuclear magnetic resonance (NMR) spectra of CTV. Early reports established that CTV was fluorescent [7,22].

CTV has been extracted from commodities and fungal cultures with a variety of solvents, including ethanol (used in the early toxicity testing), chloroform [13,17], dichloromethane [21] and aqueous methanol [23,24,25]. Before 1988 most analytical methods for detecting CTV relied upon thin-layer chromatography, however, in that year a normal phase liquid-chromatography method with fluorescence detection (LC-FLD) was reported [21]. With a mobile phase of ethyl acetate-hexane (3+1) the fluorescence was detected at 480 nm using an excitation of 388 nm. More recently, normal phase LC-FLD was used to detect CTV in rice [4]. CTV has also been measured using reverse phase LC with photodiode array detection (LC-PDA) [13]. Several laboratories have reported reverse phase LC-tandem mass spectrometry (LC-MS/MS) methods [13]. The molecular ion in positive mode was observed at *m/z* 403.2, with the main fragments at *m/z* 315 and 139 [13,18]. Detailed mass spectra were reported by Rebuffat et al. [26].

The total synthesis of CTV gave a product that upon exposure to ambient light yielded a mixture of two major components. This led to the realization authentic CTV, which exists as the all-trans form, undergoes photoisomerization, with the product termed “iso-CTV” [27]. The ratio of CTV:iso-CTV has been reported as 7:3 [28] and 3:2 [4,27]. Handling the purified CTV only under red light minimized the isomerization, however handling the toxin under typical laboratory ambient light resulted in the mixture reaching a photostable state within only 1 to 9 h [27]. Even when stored under frozen conditions and protected from light, CTV has been reported to isomerize [13]. As such it should be considered that, under typical laboratory conditions, preparations of “citreoviridin” will likely exist as mixtures that at equilibrium have ratios of CTV:iso-CTV ranging from 1.5:1 to 2.3:1.

Antibodies against CTV have been reported previously. These included polyclonal antibodies [23], a monoclonal antibody (mAb) [24], and a single chain variable fragment (scFv) antibody [25]. While these represented important efforts, the sensitivities of the immunoassays allowed for improvement. In this report we undertook to develop monoclonal antibodies for the detection of CTV, to improve immunoassays for CTV, and to apply such immunoassays to spiked white rice. In an attempt to yield improved antibodies, CTV-protein conjugates were prepared using a different approach from that described previously. Following the development of the mAbs, an interesting effect of the antibodies on the fluorescence of CTV was observed and was likewise studied.

## 2. Results and Discussion

### 2.1. Antibody Development, and Tolerance to Solvents

Because of its low molecular weight, CTV was first conjugated to bovine serum albumin (BSA) prior to immunization of mice. To facilitate the identification of CTV-binding antibodies, an ovalbumin (OVA) conjugate was also prepared and used as the immobilized antigen in an indirect competitive enzyme-linked immunosorbent assay (CI-ELISA). Previous attempts to produce CTV antibodies either oxidized the hydroxy groups on CTV and then linked them to proteins [23], or formed hemisuccinate derivatives that were then linked to the proteins [24]. Both routes involved linkage through the hydroxy groups of CTV. In our attempt, the linkage was also made through the hydroxy groups, but using a carbodiimide-based chemistry that did not involve oxidation of the hydroxy groups first or require the introduction of a long linker group. When reacted with hydroxy groups, the reagent used, 1, 1′-carbonyldiimidazole, forms a carbamate linkage with primary amines of the proteins, adding only one carbon to the length of the linkage [29]. The BSA conjugate (CTV-BSA3) was administered to 10 mice and their sera was evaluated for binding to the OVA conjugate (CTV-OVA2) and for response to free CTV. Two of the sera showed binding to the immobilized antigen (CTV-OVA2) and were selected for sacrifice and splenocyte fusions. No viable products were collected from the first fusion, while the second fusion yielded only five products. Of the five, three were able to bind free CTV. Attempts to subclone these three yielded two stable cell lines designated herein as “2-2” and “2-4”. Antibodies produced by these two cell lines were evaluated in a CI-ELISA format for their sensitivity towards free CTV, cross-reactivity towards small molecules with similar structures, tolerance to solvents, and ability to detect CTV in spiked rice.

**Figure 2:**
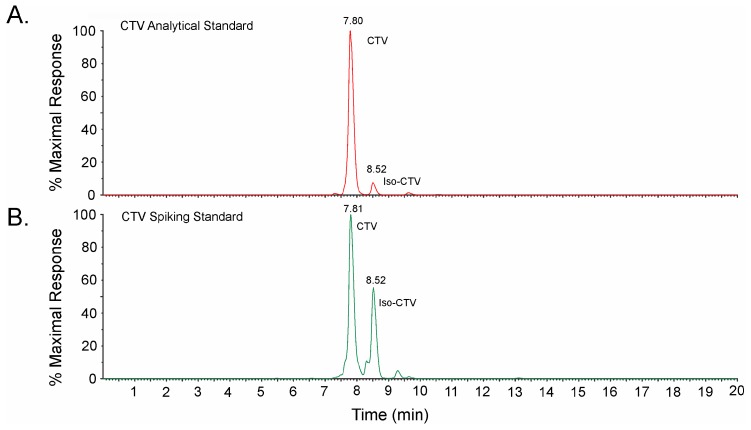
Liquid chromatography-tandem mass spectrometry (LC-MS) traces of protonated CTV and iso-CTV (*m*/*z* = 403.2). (**A**) The analytical standard used for establishing the response of the competitive enzyme-linked immunosorbent assays (CI-ELISAs) under various solvent conditions (93% CTV, 7% iso-CTV). (**B**) The standard mixture used for examining cross-reactivity to iso-CTV and for spiking rice samples (64% CTV, 36% iso-CTV).

The antibodies were relatively sensitive to CTV in buffer (0.1% OVA-phosphate buffered saline (PBS)), with IC_50_s in the range of 11 to 18 ng/mL. The analytical standard used to determine the IC_50_s contained predominantly CTV, with a small proportion of iso-CTV (7%, Figure 2A).

Three compounds having structures that resembled portions of CTV were examined for cross reactivity, including 4,6-dimethyl-α-pyrone (DMP), iso-dehydracetic acid (IDHA), and L-(+)-threose (Figure 1). DMP and IDHA have some similarity to the lactone portion of CTV. L-(+)-threose has some similarity to portions of the substituted furan of CTV. Neither IDHA nor L-(+)-threose were recognized by either of the mAbs, even at levels as high as 500 µg/mL, indicating a cross-reactivity of less than 0.004%. There was a slight inhibition with DMP at 500 µg/mL, indicating a slight cross reaction on the order of 0.04%. Obtaining IC_50_ data for DMP would have required concentrations above the solubility limit for this compound in the test buffer, hence the upper limit on the cross-reactivity. Of course, the most interesting compound to test for cross-reactivity was iso-CTV. This material was not available commercially as an analytical standard, likely because of the photoconversion between iso-CTV and CTV. However, some insights were obtained by comparing responses of standard curves prepared with a small proportion of iso-CTV (7%, Figure 2A) to those prepared with a higher proportion of iso-CTV (36%) (Figure 2B). The comparison was made by accounting for the total of the CTV and iso-CTV present. As indicated in Figures S1 and S2, when the total of CTV and iso-CTV was accounted for, the calibration curves obtained with both mixtures were nearly superimposable, with nearly identical IC_50_s. Put another way, if the calibration curves were not corrected for the presence of the iso-CTV, the preparation in Figure 2B (high iso-CTV) gave a much better ELISA response than the preparation in Figure 2A (low iso-CTV). The strong similarities between the two curves, when iso-CTV was accounted for, provided indirect evidence that the antibodies also recognize iso-CTV. Unfortunately, the absence of a relatively pure iso-CTV standard prevented us from establishing this directly.

Citreoviridin is relatively hydrophobic, with a predicted Log P of 3.04, compared to 2.68 for toluene and 3.94 for n-hexane [30]. Because of this, the conditions to extract CTV and iso-CTV from commodities use organic solvents or aqueous mixtures of organic solvents. For this reason, it was important to determine the impact of solvent concentration upon the CI-ELISAs of the two antibodies. Both antibodies demonstrated good tolerance to methanol and poorer tolerance to acetonitrile (Table 1). There was not a significant impact of methanol until the concentration used to prepare the standards reached 30% (*v*/*v*). At that point, the solubility of the OVA in the test buffer began to fail, and the variability amongst replicates worsened. The proteins began to precipitate at a lower concentration with acetonitrile (20%), which limited the concentration tested here to 15%. For the best results the methanol concentration should be kept at or below 20% and the acetonitrile concentration at or below 10%.

As noted previously, the mAbs 2-2 and 2-4 are not the first antibodies that have been applied to immunoassays for CTV detection. The development of mouse polyclonal antibodies (pAb), mouse mAbs, and a single chain variable fragment (scFv) antibody were reported by researchers at the Fujian Agricuture and Forestry University [23,24,25]. These are the only known reports of CTV antibodies, and the response of the mAbs 2-2 and 2-4 compared well to them, with lower IC_50_s and working ranges (Table 2).

### 2.2. Application of mAb 2-4 Competitive Enzyme-Linked Immunosorbent Assay (CI-ELISA) to Spiked Rice

CTV has been extracted from commodities using a wide range of solvents, from pure dichloromethane to 5% methanol [13,17,23,24,25]. To ensure compatibility with the ELISAs, a mixture of 80% methanol/20% water was used for the extractions reported here. The extracts were filtered and diluted 1:8 yielding test solutions containing 10% methanol, a level within the range that was found to be acceptable in the solvent-tolerance tests (Table 1). Yellow rice has typically been described as rice that has molded following dehulling and polishing. For this reason, the rice used for the recovery experiments was polished, dehulled, white rice. Because it was also expected that yellow rice samples would likely have been exposed to ambient lighting, and CTV is known to form an equilibrium with iso-CTV, a spiking solution was chosen that contained both CTV and significant iso-CTV (Figure 2B). Previous literature indicated that under ambient lighting CTV and iso-CTV reach an equilibrium with a ratio of approximately 1.5:1 to 2.3:1 CTV:iso-CTV. In the recovery studies reported here, the ratio of CTV:iso-CTV was 1.8:1. Rice was spiked with this mixture over the range of 0.36 to 7.24 mg/kg “total” (CTV + iso-CTV). Matrix matched calibration with the same stock solution was used to quantify the concentrations of total CTV + iso-CTV in the spiked rice.

**Figure 3:**
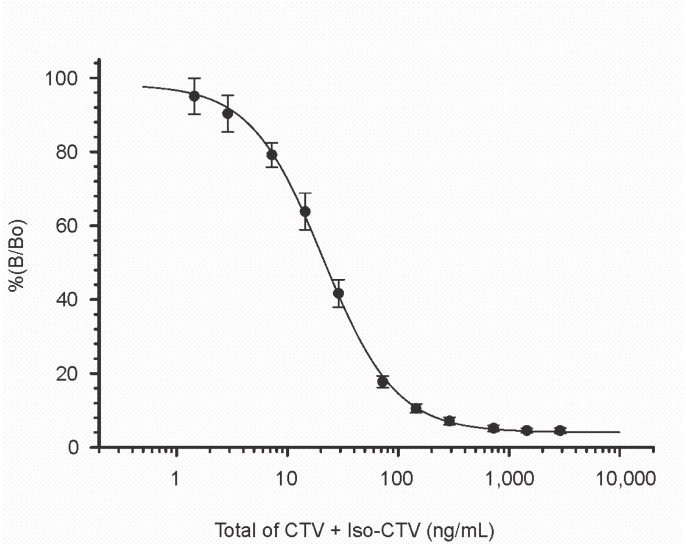
Calibration curve in rice matrix. The standard used for this curve contained a mixture of CTV and iso-CTV in the ratio of 1.8:1. Data are the mean values from 6 replicate plates with mAb 2-4. Error bars are ± 1 standard deviation (SD). The midpoint was 21.7 ng/mL, equivalent to 694 µg/kg in rice.

While mAb 2-2 was the more sensitive of the two antibodies, in terms of IC_50_s (Table 1, Figure S2), it also tended to give greater variability in response when toxin was absent (data not shown). For this reason, mAb 2-4 was selected as the basis of the screening assay for rice. Performance characteristics such as the limit of detection (LOD), IC_20_, IC_50_, and IC_80_ were determined from the calibration curve (Figure 3).

The LOD, calculated as the response three standard deviations below the response of the toxin-free samples, was estimated to be 0.13 mg/kg. The IC_20_ and IC_80_ were used to establish the dynamic range of 0.23 to 2.22 mg/kg. To improve quantification, spiked samples that contained greater than 2 mg/kg were additionally diluted to keep the resulting signals within the dynamic range. The recoveries observed were excellent (Table 3). From 30 samples covering the range of five spiking levels, the recoveries averaged 96.7% with individual recoveries that ranged from 74.0% to 118.8%. The relative standard deviation was 10.2%. We were not able to obtain any naturally contaminated samples of yellow rice that would permit evaluation beyond our recovery studies. CTV has been found at levels of up to 254 µg/kg in rice bran [4] which is at the lower end of the dynamic range, and in maize kernels at up to 2790 µg/kg [16], which is above the dynamic range of the mAb 2-4 CI-ELISA. Currently there are no regulatory levels established for CTV in commodities or foods, which makes determining a target level at which the assay should perform difficult. However, the data from oral toxicity testing provide a context for what levels may be important in foods. Recently a neurogenesis model was described wherein maternal mice were exposed to CTV through the diet from gestation day 6 through postnatal day 21. Male offspring were analyzed for effects on hippocampal neurogenesis. The no-observed-adverse-effect level was determined to be 1 mg/kg [31]. This level fits within the dynamic range for the CI-ELISA (0.23 to 2.22 mg/kg). Because of this, it appears that the CI-ELISA would be applicable to the screening of CTV and iso-CTV at levels that are toxicologically relevant.

Previous immunoassays for CTV have also reported good recoveries from rice and corn. In those experiments, the extraction solution was either 5% methanol (for rice powder) [23], or 10% to 20% methanol (for corn) [24,25]. Unfortunately, in all three cases the authors reported the spiking levels in units of “µg/mL”, rather than in units related to the mass of the food (i.e., µg/kg or mg/kg). The units of µg/mL suggested that perhaps these referred to the spiking levels in liquid extracts, rather than to spiking levels in the solid food, a point that is unclear in the manuscripts. Because of this uncertainty, it is unclear what the real spiking ranges were in the previous work. Despite this, results from all three articles suggest recoveries were good, with average recoveries of 90% [24] 84–90% [25] and 70.5–95% [23]. Given the greater IC_50_s for the previously reported antibodies (Table 2), it is likely that, on a weight basis, the spiking levels were generally higher than reported here.

### 2.3. Effects of mAbs on the Fluorescence of Citreoviridin (CTV)

**Figure 4:**
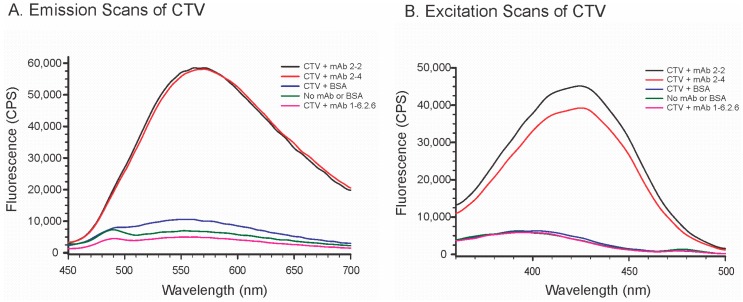
Effects of two CTV mAbs, bovine serum albumin (BSA), and a control mAb on the fluorescence of CTV. (**A**) Emission spectra with excitation at 420 nm. (**B**) Excitation spectra with emission at 570 nm. The concentration of CTV was 1.25 µM, while the concentrations of the proteins were 2.0 µM. The black and red lines represent CTV incubated with mAb 2-2 and mAb 2-4, respectively. The pink line represents the control of CTV incubated with an anti-deoxynivalenol mouse mAb (mAb 1-6.2.6). The green line represents the control of CTV in buffer without added mAb or BSA.

Molecular oxygen is known to quench most fluorophores [32]. Local environments that exclude molecular oxygen, for example the binding of a fluorophore within a protein, can increase fluorescence by reducing the inhibition caused by molecular oxygen. The previous sections have established that the mAbs selectively bound CTV. Therefore, we examined whether the fluorescence of CTV was influenced by the presence of mAbs or BSA. The concentrations of CTV and protein chosen for these experiments (1.25 and 2.0 µM, respectively) were selected so as to minimize interference from the fluorescence from the proteins themselves. The results demonstrated that the CTV mAbs significantly enhanced the fluorescence of CTV in aqueous systems (Figure 4).

Both the excitation and emission of CTV were manifest as broad peaks. The excitation maximum was 420 nm and the emission maximum 570 nm (Figure 4). BSA also enhanced the fluorescence of CTV slightly. The concentrations of the proteins used in our experiments were relatively low. For immunoglobulin Gs 2.0 µM equates to approximately 0.3 g/L, while for BSA it equates to approximately 0.13 g/L. For comparison, the reference range for human serum albumin in blood is 35–50 g/L (approximately 525 to 750 µM), which is substantially higher. At those levels we suspect that the binding of CTV would also be increased significantly, although quantifying this effect would require separating out the fluorescence of the CTV from the previously reported fluorescence of the serum albumin [33]. With regard to the antibodies, the enhancement of CTV fluorescence was seen at levels at which the fluorescence of the antibody did not interfere (e.g., Figure 4). This observation could potentially be used to determine the strength of the binding interaction, something that we have only approximated here through competitive immunoassay. The effect might also be used to establish a fluorescence-based immunoassay for CTV, analogous to systems that have been reported for ochratoxin A and zearalenone [34].

## 3. Conclusions

Two mAbs that recognize CTV were developed and applied in CI-ELISAs to the detection of CTV and iso-CTV. With CTV in buffer, the assays were of good sensitivity, with IC_50_s of 11 ng/mL (mAb 2-2) and 18 ng/mL (mAb 2-4). Assays based upon both mAbs were relatively tolerant to methanol and acetonitrile. For best results, it is recommended to keep the methanol concentration at or below 20% and the acetonitrile concentration at or below 10%. One of the mAbs (2-4) was applied to the detection of CTV and iso-CTV in spiked rice. Using matrix-matched calibration and a mixed CTV/iso-CTV standard, the dynamic range was equivalent to 0.23 to 2.22 mg/kg in rice. Recoveries were excellent, averaging 97 ± 10% over the range of 0.36 to 7.23 mg/kg. The developed mAbs and CI-ELISAs will be useful for the screening of CTV and iso-CTV in white rice. In addition, it was observed that both mAbs significantly enhanced the fluorescence of CTV, a phenomenon that may be useful in future efforts to determine the affinity of the antibodies for the toxin.

## 4. Materials and Methods

### 4.1. Materials

Except where noted otherwise, deionized water (Nanopure II, Thermo Scientific, Waltham, MA, USA) was used in the preparation of all reagents. The primary analytical standard of CTV was produced by Hayashi Pure Chemical Ind., Ltd. (Osaka, Japan). For spiking of rice samples, a standard material containing both CTV and iso-CTV prepared at the USDA-NCAUR (Peoria, IL, USA) was used [21]. Acetonitrile and methanol were HPLC grade and were purchased from Fisher Scientific (Hampton, NH, USA), as was polyvinyl alcohol (PVA). 1-1′carbonyldiimidazole (CDI), 4,6-dimethyl-2-oxo-2H-pyran-5-carboxylic acid (IDHA), 4,6-dimethyl-α-pyrone (DMP), and threose were purchased from Sigma-Aldrich (St. Louis, MO, USA). All other chemicals were reagent grade or better and purchased from major suppliers.

### 4.2. Liquid Chromatography-Photodiode Array-Mass Spectrometry (LC-PDA-MS) of CTV Stock Solutions

Two concentrated stock solutions containing predominantly CTV were prepared and analyzed by liquid chromatography-photodiode array-mass spectrometry (LC-PDA-MS) to determine relative purity. The first of the materials was prepared commercially and was used as the primary analytical standard. The second was prepared from material isolated by the late Robert Stubblefield at USDA-NCAUR (Peoria, IL, USA) and had been stored for many years at −20 °C. The latter was used to study the potential cross-reactivity of iso-CTV and was used to spike rice samples. A stock solution of the analytical standard was prepared in acetonitrile and a portion was diluted in methanol to a concentration of approximately 10 µg/mL. The ultraviolet–visible (UV–Vis) absorption spectrum was obtained using an Evolution 201 UV-Visible spectrometer (Thermo Scientific, Waltham, MA, USA). The absorbance at 383 nm was used to determine the concentration of total CTV and iso-CTV based upon the molar extinction coefficient of 44,000 [20]. A stock solution of the material used for spiking rice was prepared at nominally 0.6 mg/mL in acetone. The concentrations of CTV and iso-CTV in the spiking material were determined following LC-PDA-MS and were based upon the aforementioned analytical standard.

The LC instrument was a Dionex Ultimate 3000 system (ThermoFisher, Waltham, MA, USA). The column was a Kinetex 1.7 µ XB-C18, 100 Å, 100 × 3.0 mm, with a C18 Security Guard Ultra column (Phenomenex, Torrance, CA, USA) maintained at 30 °C. The mobile phase consisted of (A) acetonitrile, and (B) a mixture of 8% acetonitrile in water containing 0.25% acetic acid, with pH adjusted to 4 with ammonium hydroxide. The gradient began as 80% B for 4 min, then 65% B for 9 min. At 13 min the initial condition of 80% B was reinstated and the column was allowed to equilibrate for 7 min before injecting the next sample. For injection, the standards were diluted to 2 µg/mL. Injection volume was 20 µL. Flow rate was 0.7 mL/min, of which approximately 0.4 mL/min was directed to the PDA detector and 0.3 mL/min to the MS. The MS was a model QDa single quadrupole instrument (Waters Corp., Milford, MA, USA) operated in positive ion mode under the following parameters. Single ion monitoring at *m/z* = 403.2, cone voltage 6.0 V, probe temperature 425 °C, detector gain 1, capillary voltage 1.5 kV, sampling frequency 8 Hz. Under these conditions, CTV eluted at 7.8 min. At 8.5 min a second peak, with the same absorption spectrum as CTV eluted. In previous literature this peak has been termed iso-CTV, a convention also used here. Because no standards for iso-CTV were available the concentration of iso-CTV was calculated relative to that of the CTV analytical standard under the assumption that the ionization efficiencies for the two isomers were the same.

### 4.3. Preparation of CTV–Protein Conjugates

Purified CTV was linked to two proteins. One was used as a soluble antigen for immunizing mice and the other was used as an immobilized antigen in indirect ELISAs. The immobilized antigen, CTV-OVA2, was prepared as follows. All reactions were performed under conditions of reduced ambient lighting. CTV (5 mg) was dissolved in 0.25 mL acetone and 50 mg CDI was added and held at ambient temperature for 1 h. Twenty µL of water was added followed by 3.4 mL of OVA solution (50 mg OVA in 0.1 M NaHCO_3_ buffer, pH 8.6). The mixture was shielded from light and stirred for 28 h at 4 °C. The product, CTV-OVA2, was dialyzed extensively against NaHCO_3_ and phosphate buffers, using a 14 kDa membrane and culminating with 0.1 M PBS. The conjugate was diluted to a concentration of 2 mg/mL, freeze-dried, and stored at −80 °C until use. The immunogen, CTV-BSA3, was prepared in a similar fashion. However, instead of OVA the protein was BSA with a short ethylene diamine (EDA) linker attached. The preparation of EDA-BSA has been reported elsewhere [35].

### 4.4. Immunization of Animals and Isolation of mAb-Producing Clones

Immunization of mice, splenocyte fusions, cloning operations and antibody production were conducted at Envigo (Madison, WI, USA). Screening of sera and of fusion products were conducted at USDA-NCAUR (Peoria, IL, USA). All animal procedures were approved by the Institutional Animal Care and Use Committee (IACUC) of Envigo. Work was performed according to protocols 421-09a – Polyclonal Antibody Production and Hybridoma Development (approved 14 December 2018) and 638-18 Monoclonal Antibody Production (approved 14 December 2018). These protocols were developed in accordance with guidelines established by the U.S. National Institutes of Health- Office of Laboratory Animal Welfare. Ten female Balb/C mice were given a primary immunization of 100 µg CTV-BSA3 using the same procedures as described previously for production of paxilline antibodies [36]. Mouse antisera and hybridoma culture supernatant solutions were tested by competitive indirect ELISA (CI-ELISA). In initial experiments 0.1 mL of CTV-OVA2 (10 µg/mL in 0.05 M sodium phosphate buffer, pH 7.2), was incubated in wells of polystyrene microtiter plates overnight at 4 °C. The plates were washed twice with 0.32 mL Tween-PBS (0.02% *v*/*v* Tween-20 in 0.01 M PBS, pH 7.2) and blocked with 0.32 mL of PVA-PBS (1% *w*/*v* PVA in 0.01 M PBS) for 2 h at ambient temperature. Test solutions were prepared by mixing equal volumes of toxin standard solution (or control solution) and antiserum (alternatively, culture supernatant) diluted in BSA-PBS (1% *w*/*v* BSA in 0.01 M PBS) in the wells of a polypropylene microtiter plate (Corning Inc., Corning, NY, USA). The test plate containing immobilized antigen was washed twice with Tween-PBS and 0.1 mL of test solution was transferred to each well and incubated for 30 min at ambient temperature. The plate was then washed three times and 0.1 mL of a 1:2000 dilution of goat anti-mouse peroxidase conjugate was added. After incubating at ambient temperature for 30 min the plate was washed four times and the substrate o-phenylenediamine (OPD) was added. Preparation of the substrate is described elsewhere [36]. After 5 min 0.1 mL of 1 N hydrochloric acid was added to stop the reaction. Color development was determined by measuring the absorbance at 490 nm using a Synergy Neo microplate reader (Bio-Tek, Winooski, VT, USA).

From the 10 mice two were selected to undergo splenocyte fusion. The animals were sacrificed and the spleens aseptically removed. The splenocytes were chemically fused with NS-1 myeloma cells using polyethylene glycol then plated in HAT selection media. After 10 days, cultures were isolated and screened for anti-CTV activity using the CI-ELISA described earlier in this section. The first fusion yielded only a single product, which did not recognize free CTV. The second fusion also yielded a low number of products (5) of which three recognized free CTV. From these three products two clones were subsequently isolated, expanded, and used to produce larger amounts of antibody for evaluation. The cell lines were designated 2-2.2.2.8 (herein referred to as “2-2”) and 2-4.3.5.1.2.1.4 (herein referred to as “2-4”). Ascites fluid from mice administered these cell lines was partially purified by ammonium sulfate precipitation using procedures described previously [37], then dialyzed against 0.1 M PBS and freeze-dried. The protein content was determined using the BCA Protein Assay (Thermo Fisher, Waltham, MA, USA).

### 4.5. Effects of Methanol and Acetonitrile on Two CTV mAbs

The impact of methanol and acetonitrile on the CI-ELISAs with mAbs 2-2 and 2-4 was determined by incorporating the solvents at concentrations from 5% to 30% (*v*/*v*) in the diluent used to prepare the calibration curves. The analytical CTV stock solution (i.e., 93% CTV, 7% iso-CTV) was used for these experiments. CI-ELISAs were performed essentially as in Section 4.4, with a lower concentration of CTV-OVA2 immobilized (4 µg/mL). Preliminary experiments indicated that using mAb 2-2 at a concentration of 12 µg/mL or mAb 2-4 at a concentration of 4.9 µg/mL were sufficient to give color development of approximately 1 absorbance unit with a 5 min substrate incubation. As described in Section 4.4 the antibodies (in 0.1% OVA-PBS) were mixed with the toxin/solvent combinations 1+1 before transferring them to the test plates containing immobilized CTV-OVA2. Note that at 30% methanol, the OVA was very poorly soluble and with 20% acetonitrile it was insoluble. For these reasons the highest solvent concentrations tested were 30% methanol and 15% acetonitrile. Tests were conducted with 4 to 8 replicate microtiter plates. Each CTV concentration level was represented by 4 wells on the plate. To obtain the most accurate comparisons, the two antibodies were compared side-by-side on the same plates. Standards were prepared over the range of 0.2 to 1000 ng/mL. Absorbance of all samples (i.e., “B”) were normalized to those of the toxin-free controls (i.e., “Bo”) using the equation (B/Bo) × 100%. Calibration curves based upon the transformed data were obtained using a logistic dose-response model and curve fitting software (TableCurve curve 2D, Systat Software Inc., Richmond, CA, USA). Fitted curves were used to calculate the concentrations required to inhibit color development by 50% (IC_50_s).

### 4.6. Spiking and Extraction of Rice Samples

Polished long-grain white rice was kindly supplied by Susan P. McCormick (USDA-ARS-NCAUR). The rice had been milled so the husk, bran, and germ were removed. The rice was ground in a coffee mill to produce a powder similar in consistency to flour. Ten g of ground rice were spiked with a mixture of CTV and iso-CTV. The spiking solution contained a total of 144.7 µg/mL in methanol, of which 93 µg/mL was CTV and 51.7 µg/mL was iso-CTV. The volumes, amounts, and corresponding levels of CTV and iso-CTV in the rice are summarized in Table 4. Six replicate samples were produced at each spiking level. Samples were kept overnight at ambient temperature to permit the solvent in the spiking solution to evaporate. Samples were extracted with 40 mL of 80% (*v*/*v*) methanol/water by shaking for 2 h on a Burrell model 75 wrist-action shaker (Burrell Corporation, Pittsburgh, PA, USA) for 2 h at ambient temperature. The mixture was filtered through a Whatman 2V filter (Whatman plc, Maidstone, UK). The filtrate was diluted 1+7 (*v*/*v*) with a solution of 1% OVA in 10 mM PBS. The resulting diluted extracts contained 0.03125 g equivalents of rice/mL. Unspiked, control, rice was likewise extracted, diluted, and the diluted extract was used to prepare matrix-matched calibration curves for use in the CI-ELISA experiments.

### 4.7. CI-ELISA of Rice Samples

Samples of rice spiked with a mixture of CTV and iso-CTV in the ratio of 1.8:1 were compared to calibration curves prepared using the same spiking mixture. The standards were prepared by diluting the stock solution in a control rice extract consisting of 0.03125 g equivalents of rice/mL (Section 4.6). As this was a 1:8 dilution of an 80% methanol extract of rice, the diluted extract also contained 10% methanol. The concentrations of the standards, represented as the sum of CTV and iso-CTV, ranged from 1.45 ng/mL to 2894 ng/mL. Because the matrix consisted of 0.03125 g equivalents/mL, the calibration curve covered the range from 0.046 to 93 mg/kg rice. To improve quantification, the spiked samples that contained greater than 2 mg/kg were diluted further to keep the resulting signals within the dynamic range of 80% to 20% of the maximal signals (i.e., between the IC_20_ and IC_80_). The LOD of the assay in rice matrix was calculated by measuring the standard deviation of the unspiked (toxin-free) controls and calculating the CTV concentration required to observe a signal 3 standard deviations from the mean of the controls.

### 4.8. Effects of mAbs and Bovine Serum Albumin (BSA) on CTV Fluorescence

Preliminary experiments indicated that the mAbs could influence the fluorescence of CTV in aqueous buffer. To examine this further, mixtures of mAb or BSA were combined with CTV in aqueous buffer (10 mM PBS, pH 7.2). The final concentrations of mAb (or BSA) and CTV were 2.0 µM and 1.25 µM, respectively. The volumes of test solutions were 0.32 mL. Excitation and emission spectra were collected using a Neo microplate reader (BioTek, Winooski, VT, USA). Emission scans were collected using an excitation of 420 nm, with emission monitored over the range of 450 to 700 nm in 1 nm increments. Excitation scans were collected using an emission of 570 nm, with excitation provided over the range of 360 to 500 nm in 1 nm increments. Both excitation and emission data were collected with the top optics of the instrument and the following parameters: gain 150, normal reading speed, optics positioned 4.50 mm above the sample, and temperature 20.5 °C. Data from triplicate plates were averaged and imported into TableCurve. Spectra were smoothed using Savitzky–Golay filtering.

## Figures and Tables

**Table 1:** Effects of methanol and acetonitrile on the CI-ELISAs based upon mAbs 2-2 and 2-4.

Solvent	Concentration ^a^	IC_50_ for CTV (ng/mL)		Replicates ^b^
		mAb 2-2	mAb 2-4	
Buffer ^c^	-	11.1 ± 3.2	17.9 ± 5.6	8
Methanol	10%	14.8 ± 1.2	20.4 ± 0.5	4
	15%	13.7 ± 1.2	21.7 ± 4.6	6
	20%	15.2 ± 1.6	21.0 ± 1.1	4
	30%	24.9 ± 14.6	36.9 ± 12.5	4
Acetonitrile	5%	13.3 ± 2.1	20.7 ± 1.1	4
	10%	18.7 ± 3.3	29.2 ± 4.6	4
	15%	29.6 ± 2.5	43.4 ± 4.2	4

^a^ Concentration of solvent in which the CTV standards were prepared (percentage, *v*/*v*). The concentration of solvent in the competition mixture is half of this value. ^b^ Number of replicate plates used in the statistic. ^c^ 0.1% ovalbumin-phosphate buffered saline (OVA-PBS).

**Table 2:** Immunoassays for CTV.

Type of Antibody	IC_50_ for CTV (ng/mL)	Dynamic Range (IC_20_ to IC_80_) (ng/mL)	Citation
pAb	560	Not specified	[23]
mAb	161	11–2370	[24]
scFv	120	25–562	[25]
mAb	11	3–29	this work, mAb 2-2
mAb	18	5–42	this work, mAb 2-4

**Table 3:** Recovery of a mixture of CTV and iso-CTV from spiked rice.

CTV (mg/kg)	Iso-CTV (mg/kg)	Total (CTV + Iso-CTV)	Average Recovery (% ± 1 SD) ^a^	Number of Replicates ^b^
0.23	0.13	0.36	91.9 ± 14.3	6
0.46	0.26	0.72	89.6 ± 7.8	6
0.93	0.52	1.45	97.9 ± 3.2	6
1.86	1.03	2.89	106.2 ± 8.7	6
4.65	2.58	7.23	97.7 ± 7.1	6
Average (all):			96.7 ± 10.2	30

^a^ Mean recovery ± one standard deviation (SD). ^b^ Number of rice samples that were spiked.

**Table 4:** Preparation of spiked rice samples.

Volume of Stock Added (µL)	Amount of CTV Added (µg)	Amount of Iso-CTV Added (µg)	Concentration of CTV in Spiked Rice (mg/kg)	Concentration of Iso-CTV in Spiked Rice (mg/kg)	Concentration of CTV + Iso-CTV in Spiked Rice (mg/kg)
25	2.33	1.29	0.233	0.129	0.362
50	4.65	2.58	0.465	0.258	0.723
100	9.30	5.17	0.930	0.517	1.447
200	18.60	10.34	1.861	1.034	2.894
500	46.50	25.85	4.652	2.585	7.235

## Supplementary Materials

The following are available online at https://www.mdpi.com/2072-6651/11/11/630/s1, Figure S1: Effect of iso-CTV on the calibration curve in diluted rice matrix using mAb 2-4, Figure S2: Effect of iso-CTV on the calibration curve in diluted rice matrix using mAb 2-2.
