# Supplementary material for: Development and Characterization of Monoclonal Antibodies for the Mycotoxin Citreoviridin
Source: Toxins (Basel). 2019 Oct 30;11(11):630. doi: 10.3390/toxins11110630 (PMC6891493; doi:10.3390/toxins11110630)
Supplement: Supplementary file 1 [file toxins-11-00630-s001.pdf]

Supplementary Materials: Development and Characterization of Monoclonal Antibodies for the Mycotoxin Citreoviridin

Chris M. Maragos , Yosuke Uchiyama, Naoki Kobayashi, Fumichika Kominato and Yoshiko Sugita-Konishi

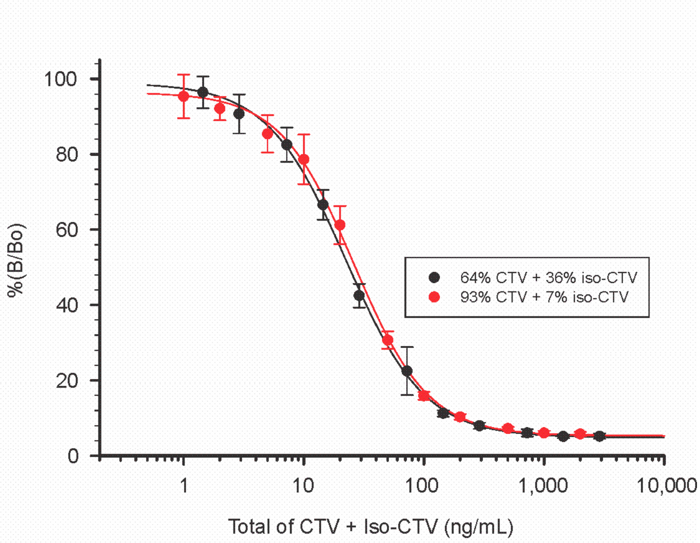

Figure S1. Effect of iso-CTV on the calibration curve in diluted rice matrix using mAb 2-4. Data are averages from 3 plates ± 1 standard deviation. IC_{50}'s were 27.0 ± 3.4 ng/mL and 23.6 ± 1.0 ng/mL for the low iso-CTV (i.e. 7%) and high iso-CTV preparation (i.e. 36%) respectively. The similar responses from the two preparations, despite their very different proportions of iso-CTV, suggested that mAb 2-4 cross-reacted with iso-CTV.

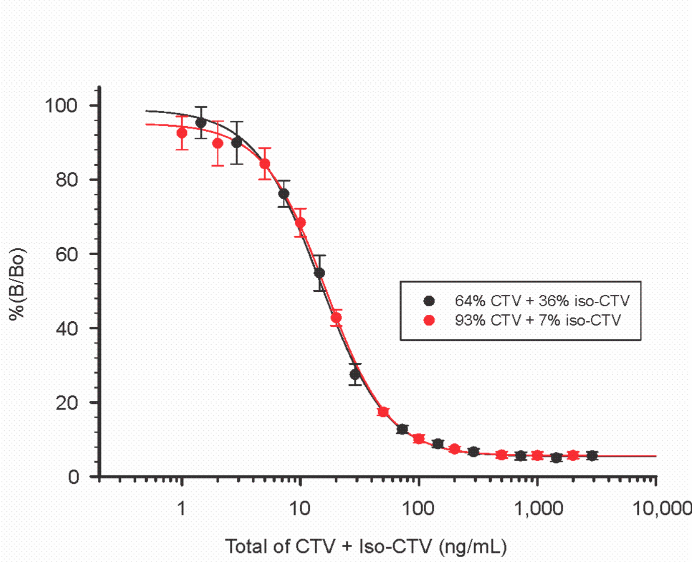

Figure S2. Effect of iso-CTV on the calibration curve in diluted rice matrix using mAb 2-2. Data are averages from 3 plates ± 1 standard deviation. IC_{50}'s were 16.5 ± 0.3 ng/mL and 15.7 ± 0.4 ng/mL for the low iso-CTV

(i.e. 7%) and high iso-CTV preparation (i.e. 36%) respectively. The similar responses from the two preparations, despite their very different proportions of iso-CTV, suggested that mAb 2-2 cross-reacted with iso-CTV.
